# Neuropsychological Profile in Pediatric Migraine without Aura: A Pilot Study

**DOI:** 10.3390/brainsci11121582

**Published:** 2021-11-29

**Authors:** Maria Esposito, Lorenzo Antinolfi, Marco Carotenuto

**Affiliations:** Dipartimento di Salute Mentale e Fisica e Medicina Preventiva, Università degli Studi della Campania “Luigi Vanvitelli”, 81100 Caserta, Italy; l.antinolfi@gmail.com (L.A.); marco.carotenuto@unicampania.it (M.C.)

**Keywords:** children, migraine without aura, NEPSY-2, neuropsychological skills

## Abstract

Despite the high prevalence of headache in developmental age, current reports about its neuropsychological effects are still lacking. The aim of the present pilot study is to assess the neuropsychological skills among children affected by migraine without aura (MwoA). Fifteen children (7M/8F) (mean age 10.73 ± 2.13) with MwoA, consecutively referred to the Center for Childhood Headache at Università degli Studi della Campania “Luigi Vanvitelli”, underwent the Italian version of the NEPSY-2 after cognitive evaluation. Moreover, to assess the pain level and disability grade during daily activity, the VAS and PedMIDAS scales were used. MwoA children were comparable with the control group of 38 children with respect to age, gender, language, and education level. Written informed consent was obtained from all parents and from children directly, when appropriate. MwoA children differed from controls significantly among the NEPSY-2 subscales, with a relevant relationship between the frequency and intensity of the attacks. In conclusion, the results of the present pilot study may suggest that MwoA could impact significantly neuropsychological functioning in children.

## 1. Background

Migraine is a common disabling primary headache disorder with relevant socioeconomic impacts during an affected individual’s lifespan [1]. Despite the high overall prevalence of migraine, limited and scarce studies have been published about neuropsychological skills, specifically in developmental age [2,3,4,5,6,7,8,9,10,11,12,13]. 

Moreover, the neurocognitive aspects in MwoA subjects are still debated independently with respect to age and gender [2,3,4,5,6,7,8,9,10,14,15,16]. 

In general, some studies have shown the presence of visuospatial memory impairment in both MwoA and migraine with aura (MwA) [5,6], and changes in verbal memory have been identified in children affected by MwoA [11]. In addition, deficits have been encountered with respect to the the recognition memory, verbal expression, and information processing speed [2,17,18]. In 2002 Calandre et al. [19] confirmed the presence of alterations in specific neuropsychological functions, such as memory, attention, and information processing, in patients affected by MwoA and MA related to general cortical hypo-perfusion, with approximately 43% of patients with impairments in visuospatial memory. Specifically, in migraineurs, differences in the cortical processing of visual stimuli compared to healthy subjects were found. These differences compromised the ability of migraineurs to identify the overall direction of motion, suggesting the purely perceptual origin of this specific altered task [19].

Moreover, during the migraine attacks, clear, unstable, and reversible cognitive impairment has been reported. In 2000, Meyer et al. [20] identified the presence of difficulty in concentration, understanding, and communication abilities during the ictal and postdromal migraine attacks. These difficulties were promptly reverted by sleep and the administration of serotonergic agonists drugs. In this light, the role of impairment in the serotonergic pathway may be suggested as the cause of these reversible cognitive deficits. In 2003, Farmer and colleagues [21] evaluated the cognitive efficiency in migraine adult subjects by testing the reaction times, attention, and visuospatial memory before, during, and after the migraine pain phase.

On the other hand, to date, conflicting results have been found regarding the neuropsychological abilities of children affected by migraine. More studies on the neuropsychological profiles of children affected by neurodevelopmental disorders were successfully conducted using the NEPSY-II evaluation [22,23,24,25]. The NEPSY-II can be considered a valid tool for studying neuropsychological profiles in developmental age and provides a flexible approach for evaluating all areas of neuropsychological interest for children [26].

In this perspective, we hypothesize that specific neuropsychological profile can be defined by identifying the typical NEPSY-II impairments in MwoA children compared to typical developing children. Therefore, the aim of present study is to assess the putative neuropsychological profile of children with MwoA according to the NEPSY-2 evaluation.

## 2. Methods

### 2.1. Participants

In total, 15 children (7 males, 8 females) affected by MwoA, consecutively referred to the Center for Childhood Headache at the Università degli Studi della Campania “Luigi Vanvitelli”, were recruited over a 12-month period on the basis of a sequential recruitment procedure from January 2016 to December 2018. The inclusion criteria were a diagnosis of Migraine without Aura (MwoA) according to the International Headache Society third edition (HIS-3) criteria [1], age between 6 and 16 years, and the availability to participate in several neuropsychological evaluation sessions. Exclusion criteria were overweight (BMI > 85th) or obesity (BMI > 95th), a history of any other type of headache different from MwoA, Intellective Quotient (IQ) ≤ 84; neurodevelopmental disorders, a history of traumatic brain injury or any other neurological or psychiatric illness, or drug or alcohol abuse or dependence during the foregoing year.

Each MwoA patient kept a journal to record the number and intensity of attacks and concomitant symptoms. 

To assess the level of pain, a visual analogue scale (VAS) was used,. The participants placed a mark on a 10 cm-long horizontal line at the appropriate distance between the two endpoints (no pain was marked as a happy smiley, and the most intense pain imaginable was marked as a hopeless smiley).

At the time of assessment, all patients were medication-free.

The neuropsychological performance of the 15 MwoA patients was compared to that of a group of 38 typical developing children (TDC) (19 males and 19 females) comparable for age, gender, race, dexterity, language, and education level who were selected randomly from the Campania Region schools. 

Informed consent was obtained from all participants, and their parents provided written informed consent. 

### 2.2. Ethical Statement

The procedures were approved by the Ethic Committee of Università degli Studi della Campania “Luigi Vanvitelli” (prot. N.0022404/i of 23 July 2021) and were in accordance with the guidelines of the Declaration of Helsinki. 

### 2.3. Wechsler Intelligence Scale for Children—Third Edition (WISC-III)

To exclude intellectual disabilities, all children from both groups (MwoA and controls) completed the Italian version of the Wechsler Intelligence Scale for Children third edition (WISC-III) [27] applicable for subjects ranging from 6 to 16 years. Specifically, the WISC-III is composed of 13 distinct subtests divided into two scales, a Verbal Scale and a Performance Scale. The six Verbal Scale tests use language-based items, whereas the seven Performance Scales use visual-motor items that are less dependent on language. Five of the subtests in each scale produce scale-specific intelligent quotients (IQs) as verbal IQ (VIQ) and performance IQ (PIQ), and the 10 subtest scores produce a Full-Scale IQ (FIQ). 

### 2.4. NEPSY_II

MwoA patients and controls underwent a full neuropsychological evaluation using the Italian version of the NEPSY–II [26]. The NEPSY–II tests were administered in the same order to all participants by a trained clinician. Participants were tested in two sessions, each lasting approximately 1.5–2 h. The time taken to complete the test battery was about 3 h. We used 31 tests of the Italian version of the NEPSY–II [26]. 

All MwoA children were examined interictally at least 3 days after the last migraine attack.

According to the *NEPSY-II Clinical and Interpretive Manual* [26], across the age groups, the internal reliability coefficients [28,29] and test-retest reliability for all NEPSY-II items could be considered, for the most part, adequate or very high [30].

The NEPSY-II can provide data about 6 main neuropsychological domains, as described in the following subscetions.

#### 2.4.1. Attention and Executive Functions

Six tests make up this domain. The *Visual Attention* test assesses visual scanning and selective visual attention abilities. The participant is required to scan an A3 sheet and mark specified visual targets among several similar distractors (face drawings).

The *Design Fluency* test assesses behavioral productivity. The participant is asked to generate unique designs by connecting up to five dots presented in a structured or random array. In the *Auditory Attention and Response Set* test, the participant listens to a series of words and points to the appropriate colored circle when they hear a target word. *Auditory Attention* evaluates selective and sustained auditory attention and requires participants to point to one colored circle when the corresponding color name is heard (i.e., touching the red circle when the word *red* is said) while ignoring other color names. *Response Set* assesses the ability to shift and maintain a new, complex response set involving the inhibition of the automatic response and alternating between matching or contrasting stimuli (i.e., touching the yellow circle when the word *red* is said, the red circle when the word *yellow* is said, and the blue circle when the word *blue* is said). *Inhibition* evaluates the ability to inhibit automatic responses and to shift between congruent or incongruent responses during the naming of visual stimuli.

The *Naming* condition requires participants to name the shape of squares and circles or the up or down direction of arrows. The *Inhibition* condition requires participants to provide the opposite naming response on the same stimuli. The *Switching* condition requires participants to provide the congruent or incongruent naming response according to the color of the stimulus. The *Clocks* test evaluates planning and organization of visuospatial perception and response, as well as the concept of time, and requires participants to read or draw the times on analogical or digital clocks. *Animal Sorting* requires participants to sort cards into two groups of four cards each using various self-initiated sorting criteria.

#### 2.4.2. Language

The language domain includes six tests. *Comprehension of Instructions* evaluates noncontextual language and requires participants to indicate a given sequence of crosses and circles of different colors in response to oral instructions of increasing syntactic complexity. In the *Speeded Naming* test, the participant is required to name a series of letters and numbers as quickly as possible, thus allowing the evaluation of the velocity of lexical retrieval and production. The *Phonological Processing* test assesses phonemic awareness with tasks requiring the phonological segmentation at the level of syllables and phonemes (i.e., substitute a syllable or a phoneme in a given word to create a new word). The *Word Generation* test requires the participant to produce as many words as possible based on a semantic or phonological criterion within 1 minute for each category. The *Repetition of Nonsense Words* test assesses phonological encoding and decoding by requiring participants to repeat nonsense words presented aloud. The *Oromotor Sequences* test assesses oromotor coordination and requires participants to repeat onomatopoeic sounds and tongue twisters five times.

#### 2.4.3. Memory and Learning

Seven NEPSY–II tests evaluate verbal and visuospatial memory abilities. Four tests have both immediate and delayed recall conditions to evaluate short- and long-term memory retrieval. The *Memory for Faces* test assesses face discrimination and recognition. The participant looks at a series of faces in an incidental learning procedure. Then, in immediate and delayed recall conditions, the participant is shown a series of three-photograph arrays from which she selects the previously presented face. *Word List Interferenc*e assesses verbal working memory, repetition, and word recall following interference. The participant is presented with two lists of words and is asked to repeat each list following its presentation. Then, the participant recalls each list in order of presentation. The *Memory for Designs* test assesses memory for the form and spatial location of novel visual material. The participant is shown a series of nonfigural abstract shapes placed on a grid, which are thereafter removed from view. In immediate or delayed recall conditions after each presentation, the participant is required to select the appropriate shapes from a set of cards and to place them onto an empty grid in the same positions as they were in the sample stimulus. *List Memory* assesses long-term verbal memory for lists of words. The participant is read a list of 15 words and asked to recall them in 7 trials before and after the presentation of a further interferential list. The *Memory for Names* test assesses visuo/verbal association learning and requires participants to learn the names of children whose faces are drawn on eight cards. *Narrative Memory* assesses story recall abilities. The participant listens to a story, and the participant is then asked to repeat it. The participant is then asked questions to elicit missing details from her recall of the story. In the *Sentence Repetition* test, participants are asked to repeat sentences of increasing complexity and length.

#### 2.4.4. Sensorimotor Functions

Three tests evaluate the motor planning and execution abilities, as well as sensorimotor coordination. The *Finger Tapping* test evaluates the motor dexterity in repetitive finger movements and motor programming in sequences of finger movements. *Imitation of Hand Postures* evaluates the ideomotor praxis abilities and requires participants to imitate, with the dominant and nondominant hand, various meaningful and meaningless hand/finger positions shown by the examiner. The *Manual Motor Sequences* test assesses manual motor coordination by requiring participants to imitate a series of rhythmic hand movement sequences demonstrated by the examiner five times.

#### 2.4.5. Social Perception

Two NEPSY–II tests, one with two separate parts, evaluate social perception. In the *verbal* items of the *Theory of Mind* test, participants are provided with verbal or pictorial descriptions of some social situations. Then, the participants are asked questions about those situations that require the understanding of the character’s point of view. The *contextual* items of the *Theory of Mind* test evaluate the capacity to understand how certain emotions are linked to given social situations and to correctly recognize the emotions that the various social settings generate. *Affect Recognition* assesses the ability to recognize emotional expressions (happiness, sadness, anger, fear, disgust, and neutral) from photographs of children’s faces in four different tasks.

Participants need to match the two faces expressing the same emotions among three or more alternatives.

#### 2.4.6. Visuospatial Processing

Six NEPSY–II tests evaluate visuospatial processing. *Design Copying* assesses the constructional abilities and requires the participants to copy two-dimension figures of increasing complexity. *Block Construction* assesses the visuospatial and visuomotor ability to reproduce three-dimensional constructions from two-dimensional drawings. *Picture Puzzles* assess the visual discrimination and perception of part–whole relationships. The participant identifies the sections on a large picture from which each of four separate small pictures were taken. The *Geometric Puzzles* test assesses the ability to identify and match geometric shapes of increasing complexity (design recognition), as well as mental rotation abilities. The *Route Finding* test assesses the knowledge of visual–spatial relations and directionality, as well as the ability to transfer a route from a simple schematic map to a more complex one. The *Arrows* test assesses the ability to judge line orientation. The participant looks at an array of arrows arranged around a target and indicates the arrows that point to the center of the target.

### 2.5. Data Analysis

The scores obtained by each MwoA and TDC participant for the NEPSY–II tests were expressed as scaled scores (*M* = 10; *SD* = 3) with respect to the normative values for the corresponding chronological age [26].

To compare the NEPSY-II results between the MwoA and TDC groups, an unpaired t-test was applied. The chi-squared test was applied to compare the genders of two groups. 

To assess the relationship between the NEPSY-II scores and MwoA characteristics among affected children, Pearson’s’ correlation test was performed.

A significance threshold of *p* < 0.05 was set for all statistical tests to maximize the probability to differentiate the neuropsychological profiles of the patients and controls.

## 3. Results

The two studied groups were statistically comparable in terms of age (10.73 ± 2.12 vs. 10.43 ± 2.02; *p* = 0.634) and gender (7M/9F vs. 19M/19F; χ^2^ = 0.047; *p* = 0.826) (Table 1).

According to the WISC-III evaluation the MwoA children and TDC group were comparable for FIQ (99.6 ± 12.3 vs. 100 ± 12.4; *p* = 0.855) (Table 1), even if the MwoA children showed peculiar distribution in PIQ and VIQ, as demonstrated in our previous study about the cognitive performance of MwoA children [13]. 

According to journal recordings from the VAS evaluation, MwoA children showed 3.01 ± 0.96 mean attacks/week and a 8.72 ± 1.04 pain level.

MwoA children showed significantly lower performance for the items inhibition in situation B (*p* = 0.029), statue (*p* ≤ 0.001), instruction understanding (*p* = 0.021), immediate memory of names (*p* = 0.003), delayed memory of names (*p* = 0.038), visuomotor accuracy (*p* = 0.001), manual motor sequences (*p* = 0.007), copy of drawings (*p* = 0.029), and geometric puzzle (*p* = 0.047) compared to the healthy controls. At the same time, the MwoA children had a significantly higher performance in the items visual attention (*p* ≤ 0.001), delayed memory of faces (*p* = 0.001), and repetition of phrases (*p* = 0.016) compared to the healthy controls (Table 2, Table 3, Table 4, Table 5, Table 6, Table 7).

According to the Pearson correlation analysis, the studied sample showed a significant relationship between the frequency and intensity of the attacks and the items statue (*p* = 0.001; *p* ≤ 0.001), delayed memory of faces (*p* ≤ 0.001; *p* = 0.001), and visual-motor accuracy (*p* = 0.024; *p* = 0.003) (Table 8, Table 9, Table 10, Table 11, Table 12, Table 13).

## 4. Discussion

The main findings of the present study can be summarized and identified in differences neuropsychological skills in children with MwoA. 

The neuropsychological aspects of migraine in children have been studied in the past, but studies have often obtained conflicting results regarding both cognitive and neuropsychological functioning. In this context, the present study attempts to define, for the first time, a unique neuropsychological profile of children suffering from migraine without aura.

Over the past decades, several authors have suggested the presence of transient deficits in cognitive function in people with migraine [2,3,17,19,31,32,33] without a specific relationship between the duration and intensity of migraine and cognitive functioning [7,8,14,34]. The present report suggests a significant relationship between the frequency and duration of attacks and inhibitory control items, highlighting the role of attacks on motor and attentional abilities. Therefore, our findings confirm the impairment of memory processing speed of the information, attention, and psychomotor skills in MwoA children as suggested by previous studies [5,17,19,31,33].

Moreover, neuroimaging reports among migraineurs adults have demonstrated the presence of a characteristic pattern of morphological abnormalities in the brain described as alterations in volume of gray matter [35,36,37,38,39]. In 2014, Rocca and colleagues [39] applied voxel-based morphometry to estimate the presence and distribution of anomalies of the gray and white matter, and the correlation with the frequency of attacks and the duration of the disease in developmental age. This study demonstrates the presence of grey matter atrophy in the temporal and frontal lobes, and an increased volume of the putamen in children and adults with migraine compared to healthy controls. Although we could not collect any data about the cortical atrophy of MwoA children in the present study, we can hypothesize that anomalies in grey matter of the fronto-temporal circuits found in MwoA children by Rocca et al. [38,39] could be linked to the executive functions and memory impairment as reported in our study sample (Table 2 and Table 4).

On the other hand, more studies have shown the close relationship between cognitive/neuropsychological abilities and sleep habits in children [40,41,42,43,44,45,46], and a recent report [47] showed a significant reduction of a slow component of the cyclic alternating pattern (CAP) in the sleep architecture of MwoA children. A generator of a slow component of CAP (A1 phases) was identified in the prefrontal cortex regions, highlighting the close relationship with the intellectual level and, more generally, with the knowledge and creative processes both in adults [48] and children [41,48,49]. From this point of view, we can read the attentional and executive function abnormalities in our sample as an effect of the A1 CAP reduction in MwoA sleep [46].

Recently, according tothe brain “connectome” study, Silvestro et al. [50] found the lack of the calcarine hub in a group of MwoA adults. This finding could explain the lower performance in visuomotor accuracy of the MwoA children in our sample.

On the other hand, we must consider some limitations of the present study, such as the small sample size and the absence of neurophysiological and/or neuroimaging exams associated with the clinical evaluations of children.

Notwithstanding these limitations, the results of this study suggest the presence of peculiar neuropsychological functioning with reference to the attentional and executive abilities in migraine-affected children compared to typical developing children. Therefore, more studies are mandatory to confirm the results and clarify the nature of neuropsychological aspects and MwoA characteristics in children.

## Figures and Tables

**Table 1:** Population study.

	Age				FIQ				Gender
	Mean	SD	Range	Skewness	Mean	SD	Range	Skewness	M/F
**MwoA**	10.733	2.125	6–13	−0.794	99.600	12.263	85–127	0.785	7/9
**TDC**	10.434	2.024	6–13	−0.587	100.289	12.412	85–124	0.680	19/19

FIQ (Full-IQ); MwoA (Migraine without Aura); TDC (Typical Developing Children).

**Table 2:** Differences in the NEPSY-II attention and executive functions domain between the two study groups.

	Attention and Executive Functions		
	MwoA	TDC	*p*
**AV-Acc**	11.533 ± 1.846	9.684 ± 1.416	<0.001
**FG-Tot**	9.600 ± 2.197	9.868 ± 1.359	0.592
**IN_A-Tem**	9.933 ± 1.710	9.763 ± 1.364	0.705
**IN_B-Tem**	9.133 ± 1.457	10.184 ± 1.557	0.029
**IN_C-Tem**	9.533 ± 1.302	9.500 ± 1.371	0.936
**RA-Tot**	10.600 ± 3.924	10.105 ± 1.521	0.507
**ST-Tot**	6.133 ± 1.767	10.105 ± 1.391	<0.001

*Visual Attention Accuracy (AV_Accur); Design Fluency (FG_Tot);* Inhibition condition A Naming, time (IN_A-Tem); Inhibition Condition B inhibition, time (IN_B-Tem); Inhibition condition C Switching, time (IN_C-Tem), Animal sorting (RA-Tot); statue (ST-Tot).

**Table 3:** Differences in the NEPSY-II language domain between the two study groups.

	Language		
	MwoA	TDC	*p*
**CI-Tot**	8.667 ± 3.039	10.184 ± 1.574	0.021
**DV-Tem**	10.600 ± 2.558	10.158 ± 1.480	0.434
**DV-Com**	9.600 ± 2.165	9.763 ± 1.283	0.735
**EF-Tot**	9.800 ± 1.740	9.711 ± 1.450	0.849
**FV-Sem**	8.733 ± 3.081	9.500 ± 1.538	0.232
**FV-Fon**	10.667 ± 2.895	10.184 ± 1.557	0.436
**RnP-Tot**	8.933 ± 2.314	9.895 ± 1.269	0.058
**SOM-Tot**	10.267 ± 2.086	10.211 ± 1.492	0.913

*Comprehension of Instructions (CI-Tot); Speeded Naming (DV-Tem, DV-Com); Phonological Processing (EF-Tot); Word Generation semantic and phonological(FV-Sem,FV-Fon); Repetition of Nonsense Words (RnP-Tot); Oromotor Sequences (SOM-Tot).*

**Table 4:** Differences in the NEPSY-II memory and learning domain between the two study groups.

	Memory and Learning		
	MwoA	TDC	*p*
**MF-Imm**	9.933 ± 2.963	9.895 ± 1.607	0.951
**MF-Dif**	11.133 ± 2.167	9.368 ± 1.303	0.001
**MF-Tot**	10.800 ± 2.833	10.053 ± 1.394	0.203
**IL-Rip**	9.400 ± 2.098	9.474 ± 1.520	0.887
**IL-Rie**	9.533 ± 2.200	9.684 ± 1.491	0.774
**MD-Imm**	9.400 ± 3.158	10.289 ± 1.469	0.166
**MD-Dif**	10.067 ± 2.604	9.474 ± 1.466	0.298
**ML-Imm**	10.133 ± 3.021	9.842 ± 1.405	0.632
**ML-Dif**	9.333 ± 3.599	9.684 ± 1.509	0.616
**ML-Tot**	10.067 ± 3.081	9.737 ± 1.309	0.584
**MNo-Imm**	8.800 ± 2.597	10.526 ± 1.409	0.003
**MNo-Dif**	9.000 ± 3.024	10.342 ± 1.547	0.038
**MNo-Tot**	9.067 ± 2.520	9.816 ± 1.333	0.164
**MNa-RieSp**	9.133 ± 2.503	9.474 ± 1.330	0.522
**MNa-Rievtot**	9.133 ± 2.134	9.263 ± 1.408	0.796
**MNa-Ric**	10.400 ± 1.682	9.947 ± 1.432	0.329
**RF-Tot**	10.733 ± 1.792	9.553 ± 1.446	0.016

*Memory for Faces (*MF-Imm, MF-Dif, MF-Tot); *Word List Interferenc*e (IL-Rip, IL-Rie); *Memory for Designs (*MD-Imm, MD-Dif); *List Memory (*ML-Imm, ML-Dif, ML-Tot); *Memory for Names (*MNo-Imm, MNo-Dif, MNo-Tot); *Narrative Memory (*MNa-RieSp, MNa-Rievtot, MNa-Ric); *Sentence Repetition* (RF-Tot).

**Table 5:** Differences in the NEPSY-II sensorimotor functions domain between the two study groups.

	Sensorimotor Functions		
	MwoA	TDC	*p*
**Tap-Tem**	9.400 ± 1.454	9.974 ± 1.479	0.207
**IP-Tot**	8.867 ± 3.399	9.763 ± 1.532	0.189
**PV-Tem**	10.133 ± 2.356	10.368 ± 1.422	0.658
**PV-Com**	7.867 ± 2.167	9.632 ± 1.478	0.001
**SMM-Tot**	8.333 ± 2.920	10.000 ± 1.433	0.007

*Finger Tapping (*Tap-Tem); *Imitation of Hand Postures (*IP-Tot); Visuo-motor precision (PV-Tem, PV-Com); *Manual Motor Sequences (*SMM-Tot).

**Table 6:** Differences in the NEPSY-II social perception domain between the two study groups.

	Social Perception		
	MwoA	TDC	*p*
**TM-Tot**	10.800 ± 2.933	10.289 ± 1.334	0.385
**RE-Tot**	9.933 ± 3.348	9.947 ± 1.251	0.982

Theory of Mind (TM-Tot); Affect Recognition (RE-Tot).

**Table 7:** Differences in the NEPSY-II visuospatial elaboration domain between the two study groups.

	Visuospatial Elaboration		
	MwoA	TDC	*p*
**CD-Gen**	8.333 ± 2.795	9.632 ± 1.422	0.029
**CD-Spe**	9.533 ± 2.416	10.079 ± 1.440	0.315
**CB-Tot**	8.867 ± 2.588	10.026 ± 1.568	0.051
**PF-Tot**	9.133 ± 2.997	10.237 ± 1.422	0.074
**PG-Tot**	8.600 ± 3.888	10.053 ± 1.355	0.047

*Design Copying* (CD-Gen, CD-Spe); *Block Construction* (CB-Tot); *Picture Puzzles* (PF-Tot); *Geometric Puzzles* (PG-Tot).

**Table 8:** Pearson’s correlation analysis results between NEPSY-II attention and executive functions parameters and MwoA clinical characteristics.

Attention and Executive Functions		
	MwoA Frequency/Month	MwoA Intensity
AV-Acc	*r* = 0.2213 *p* = 0.111	*r* = 0.3718 *p* = 0.006
FG-Tot	*r* = −0.0770 *p* = 0.584	*r* = −0.0062 *p* = 0.965
IN_A-Tem	*r* = 0.2086 *p* = 0.134	*r* = 0.1192 *p* = 0.395
IN_B-Tem	*r* = −0.1993 *p* = 0.153	*r* = −0.2215 *p* = 0.111
IN_C-Tem	*r* = 0.1138 *p* = 0.417	*r* = 0.0541 *p* = 0.701
RA-Tot	*r* = −0.0511 *p* = 0.716	*r* = 0.0839 *p* = 0.550
**ST-Tot**	***r* = −0.4315** ***p* = 0.001**	***r* = −0.6799** ***p* = 0.000**

*Visual Attention Accuracy (AV_Accur); Design Fluency (FG_Tot);* Inibition condition A Naming, time (IN_A-Tem); Inhibition Condition B inhibition, time (IN_B-Tem); Inhibition condition C Switching, time (IN_C-Tem), Animal sorting (RA-Tot); statue (ST-Tot).

**Table 9:** Pearson’s correlation analysis results between NEPSY-II language parameters and MwoA clinical characteristics.

Language		
	MwoA Frequency/Month	MwoA Intensity
CI-Tot	*r* = −0.2743 *p* = 0.047	*r* = −0.3488 *p* = 0.010
DV-Tem	*r* = −0.0124 *p* = 0.930	*r* = 0.0078 *p* = 0.956
DV-Com	*r* = −0.1345 *p* = 0.337	*r* = −0.1002 *p* = 0.475
EF-Tot	*r* = −0.1145 *p* = 0.414	*r* = −0.0714 *p* = 0.611
FV-Sem	*r* = −0.2721 *p* = 0.049	*r* = −0.1540 *p* = 0.271
FV-Fon	*r* = −0.1532 *p* = 0.274	*r* = 0.0485 *p* = 0.730
RnP-Tot	*r* = −0.0109 *p* = 0.938	*r* = −0.0864 *p* = 0.538
SOM-Tot	*r* = 0.1932 *p* = 0.166	*r* = 0.0349 *p* = 0.804

*Comprehension of Instructions* (CI-Tot); *Speeded Naming* (DV-Tem, DV-Com); *Phonological Processing* (EF-Tot); *Word Generation semantic and phonological* (FV-Sem,FV-Fon); *Repetition of Nonsense Words* (RnP-Tot); *Oromotor Sequences* (SO M-Tot).

**Table 10:** Pearson’s correlation analysis results between NEPSY-II memory and learning parameters and MwoA clinical characteristics.

Memory and Learning		
	MwoA Frequency/Month	MwoA Intensity
MF-Imm	*r* = 0.2745 *p* = 0.047	*r* = 0.1920 *p* = 0.168
**MF-Dif**	***r* = 0.4676** ***p* = 0.000**	***r* = 0.4394** ***p* = 0.001**
MF-Tot	*r* = 0.3115 *p* = 0.023	*r* = 0.3033 *p* = 0.027
IL-Rip	*r* = −0.0424 *p* = 0.763	*r* = −0.0385 *p* = 0.784
IL-Rie	*r* = −0.0709 *p* = 0.614	*r* = −0.1592 *p* = 0.255
MD-Imm	*r* = −0.0179 *p* = 0.899	*r* = −0.1826 *p* = 0.191
MD-Dif	*r* = 0.1588 *p* = 0.256	*r* = 0.1574 *p* = 0.260
ML-Imm	*r* = 0.0122 *p* = 0.931	*r* = 0.1749 *p* = 0.210
ML-Dif	*r* = −0.2290 *p* = 0.099	*r* = 0.0281 *p* = 0.841
ML-Tot	*r* = −0.0460 *p* = 0.744	*r* = 0.1766 *p* = 0.206
MNo-Imm	*r* = −0.1600 *p* = 0.252	*r* = −0.2905 *p* = 0.035
MNo-Dif	*r* = −0.1156 *p* = 0.410	*r* = −0.1238 *p* = 0.377
MNo-Tot	*r* = −0.0307 *p* = 0.827	*r* = −0.0872 *p* = 0.535
MNa-RieSp	*r* = 0.0611 *p* = 0.664	*r* = −0.1413 *p* = 0.313
MNa-Rievtot	*r* = 0.0944 *p* = 0.501	*r* = −0.1989 *p* = 0.153
MNa-Ric	*r* = −0.1175 *p* = 0.402	*r* = 0.0114 *p* = 0.935
RF-Tot	*r* = 0.1586 *p* = 0.257	*r* = 0.1491 *p* = 0.287

*Memory for Faces (*MF-Imm, MF-Dif, MF-Tot); *Word List Interferenc*e (IL-Rip, IL-Rie); *Memory for Designs (*MD-Imm, MD-Dif); *List Memory (*ML-Imm, ML-Dif, ML-Tot); *Memory for Names (*MNo-Imm, MNo-Dif, MNo-Tot); *Narrative Memory (*MNa-RieSp, MNa-Rievtot, MNa-Ric); *Sentence Repetition* (RF-Tot).

**Table 11:** Pearson’s correlation analysis results between NEPSY-II sensorimotor functions parameters and MwoA clinical characteristics.

Sensorimotor Functions		
	MwoA Frequency/Month	MwoA Intensity
Tap-Tem	*r* = −0.1779 *p* = 0.202	*r* = −0.1751 *p* = 0.210
IP-Tot	*r* = 0.0064 *p* = 0.964	*r* = 0.0035 *p* = 0.980
PV-Tem	*r* = −0.3356 *p* = 0.014	*r* = −0.1256 *p* = 0.370
**PV-Com**	***r* = −0.3108** ***p* = 0.024**	***r* = −0.3960** ***p* = 0.003**
SMM-Tot	*r* = −0.0074 *p* = 0.958	*r* = −0.1843 *p* = 0.186

*Finger Tapping (*Tap-Tem); *Imitation of Hand Postures (*IP-Tot); Visuo-motor precision (PV-Tem, PV-Com); *Manual Motor Sequences (*SMM-Tot).

**Table 12:** Pearson’s correlation analysis results between NEPSY-II social perception parameters and MwoA clinical characteristics.

Social Perception		
	MwoA Frequency/Month	MwoA Intensity
TM-Tot	*r* = −0.0676 *p* = 0.630	*r* = −0.0930 *p* = 0.508
RE-Tot	*r* = −0.1852 *p* = 0.184	*r* = −0.0898 *p* = 0.523

*Theory of Mind* (TM-Tot); *Affect Recognition* (RE-Tot).

**Table 13:** Pearson’s correlation analysis results between NEPSY-II visuospatial elaboration parameters and MwoA clinical characteristics.

Visuospatial Elaboration		
	MwoA Frequency/Month	MwoA Intensity
CD-Gen	*r* = −0.0850 *p* = 0.545	***r* = −0.3314** ***p* = 0.015**
CD-Spe	*r* = 0.0961 *p* = 0.494	*r* = −0.0940 *p* = 0.503
CB-Tot	*r* = 0.0454 *p* = 0.747	*r* = −0.2464 *p* = 0.075
PF-Tot	*r* = −0.0958 *p* = 0.495	*r* = −0.1678 *p* = 0.230
PG-Tot	*r* = −0.0620 *p* = 0.659	***r* = −0.3507** ***p* = 0.010**

*Design Copying* (CD-Gen, CD-Spe); *Block Construction* (CB-Tot); *Picture Puzzles* (PF-Tot); *Geometric Puzzles* (PG-Tot).

## Data Availability

Not applicable.
